# Variants in *SUP45* and *TRM10* Underlie Natural Variation in Translation Termination Efficiency in *Saccharomyces cerevisiae*

**DOI:** 10.1371/journal.pgen.1002211

**Published:** 2011-07-28

**Authors:** Noorossadat Torabi, Leonid Kruglyak

**Affiliations:** 1 Lewis-Sigler Institute for Integrative Genomics, Princeton University, Princeton, New Jersey, United States of America; 2 Department of Molecular Biology, Princeton University, Princeton, New Jersey, United States of America; 3 Department of Ecology and Evolutionary Biology, Princeton University, Princeton, New Jersey, United States of America; 4 Howard Hughes Medical Institute, Princeton University, Princeton, New Jersey, United States of America; The University of North Carolina at Chapel Hill, United States of America

## Abstract

Translation termination is a highly controlled process in the cell. In *Saccharomyces cerevisiae,* various regulatory factors employ genetic and epigenetic mechanisms to control this process. We used a quantitative dual luciferase reporter assay to demonstrate a difference in translation termination efficiency between two different yeast strains, BY4724 and RM11-1a. We then used a recently developed linkage mapping technique, extreme QTL mapping (X-QTL), to show that this difference is largely explained by a coding polymorphism in *TRM10* (which encodes a tRNA–methylating enzyme) and a regulatory polymorphism in *SUP45* (which encodes one of the yeast translation termination factors). BY and RM carry variants of *TRM10* and *SUP45* with opposite effects on translation termination efficiency. These variants are common among 63 diverse *S. cerevisiae* strains and are in strong linkage disequilibrium with each other. This observation suggests that selection may have favored allelic combinations of the two genes that maintain an intermediate level of translation termination efficiency. Our results also provide genetic evidence for a new role of Trm10p in translation termination efficiency.

## Author Summary

Translation, the process of protein synthesis from messenger RNA (mRNA), cannot be successfully completed without proper termination. The ends of the mRNA coding regions are marked by one of the three stop codons, which are recognized by termination factors rather than by the transfer RNAs (tRNAs) that match amino acids to the corresponding codons. Like most biological processes, translation termination is not perfect. Occasionally, tRNAs bind to stop codons, resulting in polypeptides with additional amino acids beyond the normal stop position—a phenomenon known as readthrough. Perturbations that affect the balance between termination factors and tRNAs will change readthrough. Here we demonstrate the effect of two perturbations on translation termination efficiency in the context of natural genetic variation. We show that a difference in readthrough between a laboratory and a vineyard strain of yeast is largely due to two genetic variants. One variant affects the expression level of a key translation termination factor; the other modifies the activity of a tRNA–methylating enzyme. We also show that natural selection has favored an intermediate level of readthrough.

## Introduction

Translational fidelity is essential for functional integrity of the cell. Efficient termination is an important aspect of translational fidelity, and a multitude of mechanisms participate in this highly regulated process. Translation termination in eukaryotes is mediated by two termination factors, eRF1 and eRF3 [1]. eRF1, encoded by *SUP45* in *Saccharomyces cerevisiae,* recognizes all three stop codons (UAG, UAA, and UGA) and facilitates release of the nascent polypeptide chain from the translational machinery [2], [3]. GTPase activity of eRF3, encoded by *SUP35* in *S. cerevisiae*, is required to couple the recognition of translation termination signals by eRF1 to efficient polypeptide chain release [4].

In a successful translation termination event, termination factors efficiently recognize stop codons. However, in certain instances, transfer RNAs (tRNAs) outcompete termination factors in stop codon recognition. The resulting misincorporation of an amino acid into the nascent peptide is known as translational readthrough. Therefore, during translation, any event that directly or indirectly makes a tRNA more likely to bind to a stop codon increases readthrough.

It is widely accepted that the efficiency of translation termination is modulated by both *cis*- and *trans*-acting factors [5]. In *S. cerevisiae*, the sequence surrounding the stop codon has been shown to play a major role in translation termination efficiency [6], [7]. Several *trans* factors have also been shown to affect translation termination, either directly through contacts with release factors or indirectly, as demonstrated by genetic experiments (reviewed in [8]). Moreover, recent studies of translation termination in *S. cerevisiae* have revealed genetic and epigenetic regulatory mechanisms that may enable controlled readthrough of stop codons, which can have significant effects on cellular processes such as mRNA degradation and, in some cases, can confer a beneficial phenotype to the cell [9]. The most studied example of such a mechanism is [PSI^+^], the prion conformation of the Sup35 protein, which can have pleiotropic effects on growth that vary among different yeast strains [10].

Although our knowledge of translation termination has grown in the past few decades, one can envision that many factors that modulate this complex process remain to be discovered. Natural genetic variation provides a framework for finding such factors. Linkage analysis has been successfully used to find the genetic basis of complex phenotypes in yeast at the cellular level, including growth in different chemical environments [11], sporulation efficiency [12] and growth at high temperatures [13], as well as phenotypes at the molecular level, such as genome-wide mRNA expression levels [14], [15].

Here, we employed linkage analysis to study translation termination efficiency. We used extreme QTL mapping (X-QTL) [16] to find the genetic basis for the observed difference in readthrough between two *S. cerevisiae* strains, RM11-1a (a wine strain hereafter referred to as RM) and BY4724 (a laboratory strain hereafter referred to as BY). We show that a coding polymorphism in *TRM10*, which encodes a tRNA-methylating enzyme with an unknown physiological role in the cell [17], affects readthrough in yeast. Moreover, we show that *cis*-regulatory variation that alters the expression level of *SUP45* is another factor involved in translation termination efficiency variation between BY and RM. These two yeast strains carry alleles of *TRM10* and *SUP45* with opposing effects on readthrough. The BY and RM alleles of both *TRM10* and *SUP45* are common in a diverse collection of *S. cerevisiae* strains [18] and are in significant linkage disequilibrium (LD), suggesting that readthrough may be subject to stabilizing selection.

## Results

### Dual luciferase assay reveals readthrough difference between BY and RM

In order to measure readthrough in the two parent strains, we took advantage of a dual luciferase reporter system [19]. This system uses tandem *Renilla* and firefly luciferase genes that are separated by a single in-frame stop codon. The activity of the firefly luciferase, encoded by the distal open reading frame, provides a quantitative measure of the readthrough of the stop codon that separates the two open reading frames. The activity of the *Renilla* luciferase, encoded by the proximal open reading frame, serves as an internal control for mRNA abundance. Thus, the relative abundance of these light-emitting proteins measures the efficiency of translation termination. Here, we used two separate reporters; one with UGA (stop codon) and one with CGA (sense codon) separating the *Renilla* and firefly open reading frames. For each strain, we calculated the readthrough as the ratio of firefly to *Renilla* luciferase activity in the presence of the stop codon, normalized by the observed ratio for the sense codon constructs (Table 1). We found that the readthrough in RM is higher than in BY.

**Table 1:** Readthrough measured with the dual luciferase reporter system.

Strain	Firefly/RenillaSense Codon (± SD)	Firefly/RenillaStop Codon (± SD)	Readthrough (%)
BY4724	0.633±0.045	0.00173±3.29×10^−4^	0.272±0.0409
RM11-1a	0.780±0.050	0.00381±3.65×10^−4^	0.489±0.0371

The ratio of firefly luciferase activity to the *Renilla* luciferase activity for the two reporters used are shown for BY and RM. Readthrough is calculated as the ratio of firefly to Renilla luciferase activity in the presence of the stop codon, normalized by the observed ratio for the sense codon constructs.

### Missense change in *TRM10* affects readthrough

We used X-QTL to examine the genetic basis of the readthrough difference between BY and RM in a large pool of segregants from a cross between these strains. In order to be able to select those segregants in the tails of the distribution of readthrough, we constructed a GFP reporter with a UGA stop codon inserted at the beginning of the GFP coding sequence and integrated it into the genomes of BY and RM (Materials and Methods). We also integrated an intact GFP reporter (without the stop codon) in the same position. Then, we transferred these reporters into BY and RM strains with suitable markers for X-QTL (Materials and Methods). For each strain, we calculated readthrough as the ratio of the GFP signal in the presence of the stop codon to the GFP signal in the absence of the stop codon. We showed that readthrough measured using the GFP reporter is in agreement with readthrough measured using the dual luciferase assay for both BY and RM (Table S1).

To map the genetic basis of the readthrough difference, we harvested a MAT**a** pool from a sporulation culture of BY×RM diploid hybrids containing the GFP reporter and sorted out segregants from the two extremes of the readthrough distribution by fluorescence-activated cell sorting (FACS). We selected the top 1% of the segregating population (high GFP signal) and the bottom 1% of the segregating population (low GFP signal). Samples from both selected pools as well as a sample of the whole (unselected) population were subsequently genotyped as previously described [16].

**Figure 1:**
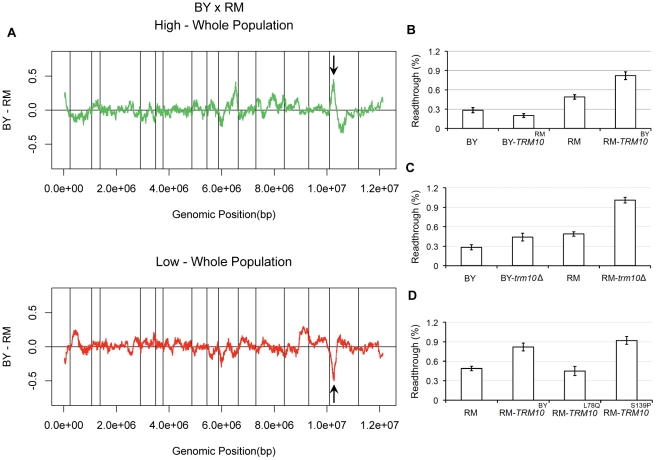
*TRM10* role in readthrough. A) X-QTL results for mapping readthrough in the BY×RM cross. The comparisons of the selected top 1% of the segregating population and the bottom 1% of the segregating population to the whole population are shown, with enrichment of the BY allele indicated by deviations above zero and enrichment of the RM allele indicated by deviations below zero. Sliding window averages (40 kb) are plotted. The black arrows show allele frequency skew on chromosome XV. B) Readthrough measured by dual luciferase assay for the parent strains and the two *TRM10* swapped strains are shown. C) Readthrough for the parent strains and *TRM10* knocked out strains are shown. D) Readthrough is shown for the RM parent strain, RM parent strain with the BY allele of *TRM10* (RM-*TRM10* ^BY^) and two RM strains made by site-directed mutagenesis RM-*TRM10* ^L78Q^ and RM-*TRM10* ^S139P^.

Comparisons of the high and low segregant pools to the whole population showed allele frequency differences on chromosome XV, at the same position but in opposite directions in the two selected tails (Figure 1A). The directions of the skew suggested that carrying a BY allele in this region results in increased readthrough, whereas carrying the RM allele results in a decrease in readthrough. Based on functional annotations available in the *Saccharomyces* Genome Database and sequence comparison between BY and RM for the genes in this region (Figure S1), we selected *TRM10* as a candidate for further investigation. *TRM10* encodes a tRNA modifying enzyme, which methylates the N-1 position of guanosine-9 in ten tRNAs in yeast [17]. Comparison of the coding sequence of *TRM10* between BY and RM showed eight single nucleotide polymorphisms (SNPs) between the two yeast strains, among which five are nonsynonymous substitutions. In order to test the causality of *TRM10* polymorphisms for the observed peak on chromosome XV, we made allele replacement strains in both BY and RM (replacing *TRM10* with the version from the other strain) and measured readthrough in the newly made strains using the dual luciferase assay. Results of this experiment showed that replacing *TRM10* in each strain with the alternative allele of this gene changed readthrough in the direction predicted from the X-QTL results. RM-*TRM10* ^BY^ showed higher readthrough than RM. BY-*TRM10* ^RM^ showed lower readthrough than BY (Figure 1B). These results showed that the effect of the *TRM10* coding polymorphism on readthrough is in the opposite direction from the difference observed in the parent strains; swapping *TRM10* increased the difference in readthrough between BY and RM. This observation suggested the presence of other polymorphic factor(s) that influence translation termination efficiency.

To further analyze the relationship between the Trm10p tRNA methylation activity and translation termination efficiency, we made complete *TRM10* deletions in both genetic backgrounds. Readthrough measurements using the dual luciferase assay showed that deleting *TRM10* in both BY and RM increases readthrough, which provides further evidence for the role of Trm10p tRNA modification in translation termination efficiency (Figure 1C). Moreover, these results suggest that BY carries a partial loss of function allele of *TRM10,* because the BY allele of *TRM10* is associated with higher readthrough. To identify the causal polymorphism, we also made strains with *TRM10* ^L78Q^ and *TRM10* ^S139P^ single amino acid changes in the RM background using site-directed mutagenesis. These two polymorphisms were chosen based on fungal protein sequence alignment from the *Saccharomyces* Genome Database, which showed that these residues are highly conserved in different yeast species. Readthrough measurements in RM-*TRM10* ^L78Q^ and RM-*TRM10* ^S139P^ identified the serine to proline substitution at position 139 as the causal polymorphism (Figure 1D).

**Figure 2:**
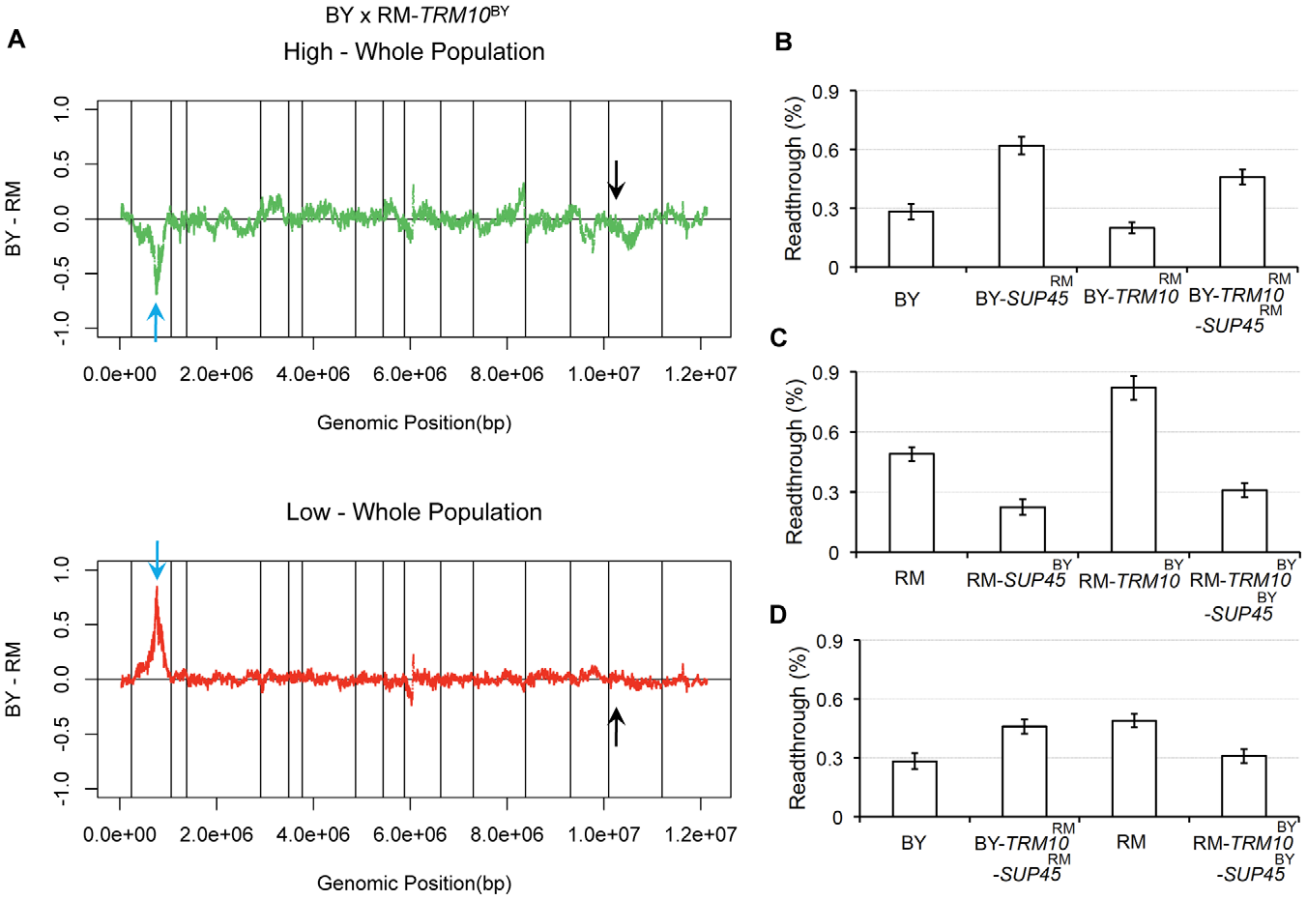
*SUP45* role in readthrough. A) X-QTL results for mapping readthrough in the *TRM10*-fixed population. The comparisons of the selected segregating population with high GFP signal and the selected segregating population with low GFP signal to the whole population are shown. Sliding window averages (40 kb) are plotted. Black arrows show *TRM10* locus on chromosome XV and blue arrows show the allele frequency skew on chromosome II. B and C) Effect of swapping *SUP45* coding and upstream sequence on readthrough is shown in B) BY and C) RM backgrounds. D) Readthrough for the parent strains, BY and RM, are compared to strains swapped for *TRM10* (coding sequence) and *SUP45* (coding and upstream sequence), BY-*TRM10* ^RM^-*SUP45* ^RM^ and RM-*TRM10* ^BY^-*SUP45* ^BY^.

To determine whether *TRM10* is the sole factor explaining the observed allele frequency skew on chromosome XV, we carried out X-QTL with segregants from a cross between BY and RM- *TRM10* ^BY^ (i.e. *TRM10* was no longer polymorphic, with both parent strains carrying the BY allele). The pool selected from the high tail of the readthrough distribution showed higher average GFP signal relative to the high tail in the original BY×RM cross (data not shown), and no allele frequency skew was observed in the *TRM10* region on chromosome XV in either selected pool (Figure 2A).

### *SUP45* expression level polymorphism contributes to readthrough difference between BY and RM

X-QTL results from the cross with both parent strains carrying the BY allele of *TRM10* showed a new region of allele frequency skew on chromosome II (Figure 2A). Our ability to detect this locus was improved by the overall increase in the GFP signal resulting from the increased readthrough conferred by the BY allele of *TRM10*. The direction of the skew on chromosome II was in the direction expected from the difference between the parent strains: the RM allele at this locus was enriched in the high-readthrough pool and depleted from the low-readthrough pool. This locus (Figure S2) contains *SUP45*, which encodes the yeast translation termination factor responsible for stop codon recognition. Sequence comparison between BY and RM showed six synonymous SNPs in the coding sequence and four nucleotide substitutions and two indels in the 400-base pair upstream noncoding region. We previously showed that expression level of *SUP45* is lower in RM (relative expression level  = 0.00333±0.0406) than in BY (relative expression level  = 0.315±0.0415) [20]. This study also showed, using an independent panel of 109 segregants, that the expression level difference mapped to the location of the *SUP45* gene. These findings strongly suggested that the observed allele frequency skew on chromosome II is due to *cis*-regulatory polymorphism that alters the expression level of *SUP45* between BY and RM.

To test this hypothesis, we made *SUP45* allele replacement strains in BY and RM, in both *TRM10*-wild type and *TRM10*-swapped backgrounds. In the first set of replacements, we swapped only the *SUP45* coding sequence. In the second set, we replaced the *SUP45* coding sequence as well as the 400-base pair upstream region. We used the dual luciferase assay to measure readthrough in the newly made strains. In both *TRM10*-wild type and *TRM10*-swapped strains, replacing the *SUP45* coding sequence along with the upstream region had a significant effect on readthrough (Figure 2B and 2C), whereas replacing just the coding sequence of *SUP45* did not have a significant effect on readthrough (Figure S3). These results support the role of *SUP45* expression level in translation termination efficiency.

**Figure 3:**
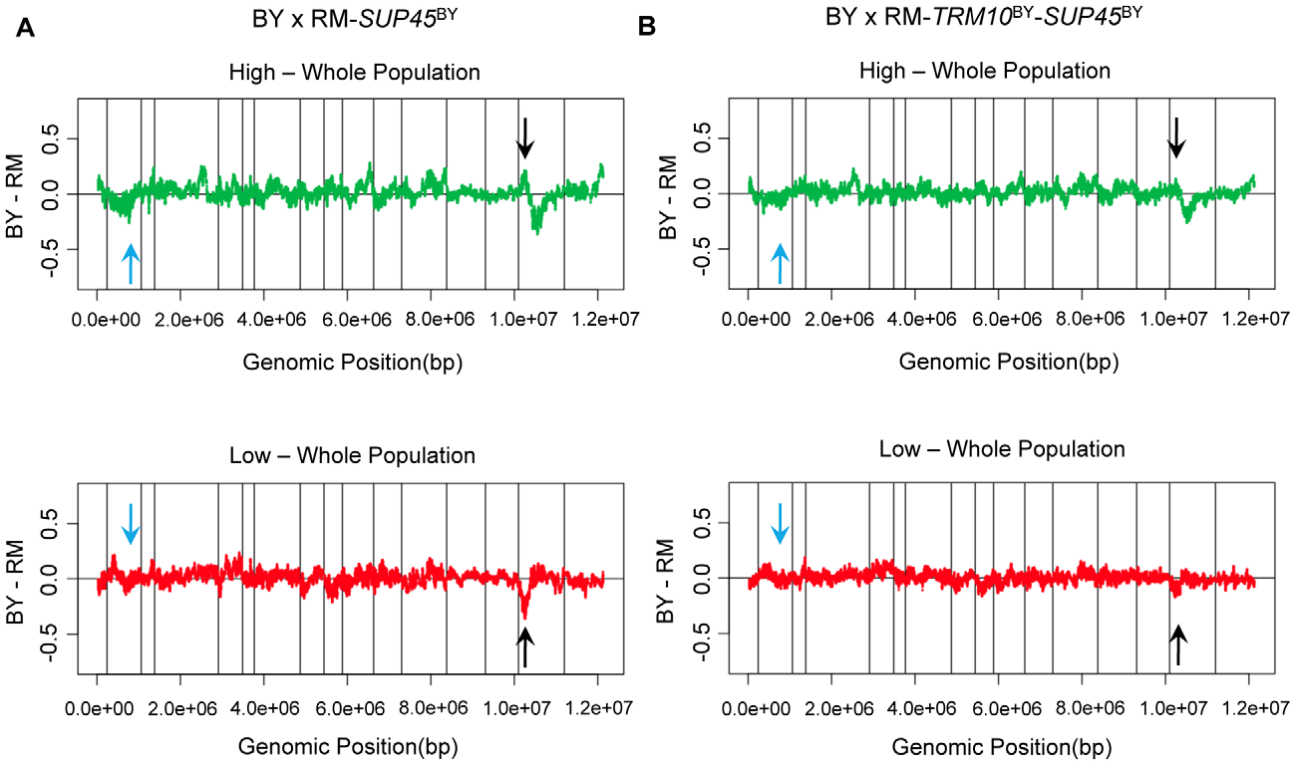
Polymorphisms in *TRM10* and *SUP45* explain readthrough difference between BY and RM. X-QTL results for mapping readthrough in A) *SUP45*-fixed population and B) *TRM10* and *SUP45*-fixed population. The comparisons of the selected segregating population with high GFP signal and the selected segregating population with low GFP signal to the whole population are shown. Sliding window averages (40 kb) are plotted. Black arrows show *TRM10* locus on chromosome XV and blue arrows show the *SUP45* locus on chromosome II.

When we swapped both *TRM10* (coding sequence) and *SUP45* (coding and upstream sequence) in each parent strain with the alleles of these genes from the other (donor) strain, translation termination efficiency changed to the level of the donor strain (Figure 2D). These data demonstrate that the polymorphisms in these two genes explain the difference in translation termination efficiency between BY and RM. We also performed X-QTL in BY×RM-*SUP45^BY^* (coding and upstream regions), as well as in BY×RM-*TRM10^BY^* (coding region)-*SUP45^BY^* (coding and upstream regions). X-QTL with the segregant pool fixed for the BY allele of *SUP45* showed the expected allele frequency skew in the region of chromosome XV containing *TRM10* (Figure 3A). X-QTL with the segregant pool fixed for the BY alleles of both *TRM10* and *SUP45* showed no significant allele frequency skews anywhere in the genome (Figure 3B), providing further evidence that these two genes explain most of the difference in translation termination efficiency between BY and RM.

### Linkage disequilibrium between *TRM10* and *SUP45*

We used a previously published polymorphism survey [18] to determine the frequencies of the BY and RM alleles of *TRM10* and *SUP45* among 63 diverse *S. cerevisiae* strains isolated from a broad range of sources. We found that both alleles of both genes are common among these *S. cerevisiae* strains, with allele frequencies of 0.46 and 0.44 for the BY alleles of *TRM10* and *SUP45*, respectively. This observation shows that these variants are not restricted to strains adapted to the laboratory environment, but rather represent naturally occurring polymorphisms in the species. Strains which carry the BY allele of *TRM10* include laboratory strains, Sake strains, clinical isolates and oak strains. Most of these strains also carry the BY allele of *SUP45* (Table S2). The *SUP45* ^BY^-*TRM10* ^BY^ and *SUP45* ^RM^-*TRM10* ^RM^ haplotypes are present in *S. cerevisiae* strains more frequently than expected based on random association (76% vs. 50%). These data suggested that *TRM10* and *SUP45* might be in linkage disequilibrium (LD).

We confirmed the presence of strong LD between these genes by calculating D' and r^2^ between *SUP45* and *TRM10* and obtained values of 0.537 and 0.266, respectively. LD could arise in part due to the population structure present in *S. cerevisiae* [18]. To test whether the observed LD is significantly higher than what is expected solely due to structure, we used polymorphisms from 63 diverse *S. cerevisiae* [18] strains and calculated LD between 1000 randomly chosen SNP pairs from different chromosomes. We observed nine pairs out of 1000 with higher r^2^ than the *TRM10*-*SUP45* pair (Figure S4), which showed that LD between *TRM10* and *SUP45* SNPs is significantly higher than what is expected due to structure alone (*p*-value  = 0.009). We obtained similar results for D' (*p*-value  = 0.011).

This non-random association of alleles suggests a functional association between the two genes. The two overrepresented haplotypes carry alleles with opposing effects on readthrough. The BY allele of *TRM10* results in higher readthrough, whereas the BY allele of *SUP45* results in lower readthrough; conversely, the RM allele of *TRM10* results in lower readthrough whereas the RM allele of *SUP45* results in higher readthrough. These two combinations keep readthrough within the range ∼0.3–0.5%, above that of the *SUP45* ^BY^-*TRM10* ^RM^ combination (∼0.2%) and below that of the *SUP45* ^RM^-*TRM10* ^BY^ combination (∼0.8%). The fact that the two allelic combinations with intermediate readthrough are overrepresented suggests that readthrough may be subject to stabilizing selection.

## Discussion

We have shown that a difference in translation termination efficiency between two yeast strains is explained by polymorphisms in two genes, *TRM10* and *SUP45*. *TRM10* encodes a tRNA-modifying enzyme, which methylates the N-1 position of guanosine-9 in some tRNAs [17]. In the *S. cerevisiae*, m^1^G is found at position 9 in 10 out of 34 tRNAs. Despite the evolutionary conservation of this modification and the conservation of the protein [17], a cellular role for m^1^G_9_ modification has not been reported. Previous studies have not found an obvious growth defect in yeast cells lacking Trm10p under standard growth conditions in rich or minimal media. Recently, a temperature-sensitive phenotype was reported in yeast cells lacking Trm10p in the presence of 5- fluorouracil [21]. This study showed that 5- fluorouracil targets tRNA-modifying enzymes and therefore reduces a number of tRNA modifications. Loss of most of the tRNA modifications in the presence of 5- fluorouracil, in combination with loss of the m^1^G_9_ modification in the absence of Trm10p, resulted in destabilization of hypomodified tRNAs, which explained the growth defect and suggested a role for m^1^G_9_ modification in tRNA stability. Here, we provide genetic evidence supporting a role for m^1^G_9_ modification in translation termination efficiency. We showed that a serine to proline substitution at position 139 of Trm10p contributes to the readthrough difference between BY and RM. One of the Trm10p substrates is the tryptophan tRNA, which decodes UGG, one of the closest codons to UGA, the opal stop codon in the dual luciferase and GFP reporters used here. According to the Pfam domain annotation, the Trm10p tRNA methylating domain spans residues 104 to 276. A serine to proline substitution at position 139 may decrease Trm10p tRNA methylation activity. Loss of this modification may increase the ability of near cognate tRNAs (such as the tryptophan tRNA) to outcompete SUP45p in UGA stop codon recognition, resulting in increased readthrough. These data support a role of tRNA methylation by Trm10p in efficient translation termination.

We also showed that the other factor involved in the translation termination efficiency difference between BY and RM is a regulatory polymorphism that alters the expression level of *SUP45*. Sup45p is the yeast translation termination factor responsible for stop codon recognition. It was previously reported that reducing the cellular level of Sup45p decreases the efficiency of translation termination [22]. When the cellular level of Sup45p is low, for example as a result of a lower expression level of *SUP45*, near cognate tRNAs are more likely to outcompete Sup45p in stop codon recognition, which in turn increases readthrough.

BY alleles of both *TRM10* and *SUP45* are common among *S. cerevisiae* strains. This observation shows that these alleles are not restricted to strains adapted to the laboratory environment, but rather represent naturally occurring polymorphisms. Despite the fact that these two genes reside on different chromosomes and are thus not physically linked, we found strong and significant linkage disequilibrium (LD) between these two genes. This non-random allelic association provides evidence that natural selection has favored the *SUP45* ^BY^-*TRM10* ^BY^ and *SUP45* ^RM^-*TRM10* ^RM^ haplotypes over the others. The preferred haplotypes consist of alleles with opposing effects on readthrough, and thus strains that carry them should exhibit intermediate readthrough relative to the other two haplotypes. This observation suggests that readthrough may be subject to stabilizing selection. High readthrough may be disadvantageous due to production of too many inappropriately extended proteins. On the other hand, keeping readthrough from dropping too low may protect yeast mRNAs whose translation requires the suppression of leaky stop codons [23].

## Materials and Methods

### Strains, media, and plasmids

Cultures were grown in minimal medium containing 0.67% (w/v) yeast nitrogen base without amino acids (Difco) containing 2% (w/v) glucose (SMD) or 2% galactose (SMGal) or 4% raffinose (SMRaf), as specified. Additional nutritional supplements or drugs were added as required. YPD plates were made as described [24]. For sporulation, SPO++ was used (http://www.genomics.princeton.edu/dunham/sporulationdissection.htm). To make a readthrough GFP reporter, a 78-nucleotide region from the *ade1-14* allele [25] was inserted in-frame with GFP coding region into HindIII site (6^th^ codon) of pGAL-GFP plasmid (kindly provided by James Broach). The insertion was confirmed by sequencing. To include a selectable marker, *NATMX* cassette was inserted into a NotI site downstream of GFP coding sequence in pGAL-GFP-*ade1-14* plasmid. To integrate this GFP reporter in yeast genome, the region containing pGAL-GFP-*ade1-14*-*NATMX* was amplified with primers with 40 base pairs of homology to regions upstream and downstream of YDL242W (an open-reading frame unlikely to encode a protein, based on available experimental and comparative sequence data from http://www.yeastgenome.org/). This PCR product was then used in transformation of BY4724 [26] and RM11-1a [14]. Insertion of the GFP reporter inside YDL242W was then confirmed by PCR. We then transferred this GFP reporter into strains with suitable genetic markers. To do so, we crossed these new BY and RM strains with GFP reporter into BY MAT**α** *can1Δ::STE2pr-SpHIS5 lyp1Δ his3Δ1* and RM MAT**α** *AMN1^BY^ his3Δ0::NatMX ho::HphMX* [16], respectively. After sporulating the obtained diploids and genotyping the dissected tetrads, we selected BY Mat**α** *his3Δ1 lyp1Δ can1Δ::STE2prSpHIS5 YDL242W::pGAL-GFP-ade1-14-NATMX* and RM Mat**a** *AMN1^BY^ his3Δ::NATMX, YDL242W::pGAL-GFP-ade1-14-NATMX* .

*TRM10* and *SUP45* replacement strains were generated by a two-step replacement method [27]. *TRM10* was replaced with *URA3-KanMX* cassette from pCORE in BY4724 and RM11-1a generating *trm10*Δ::*URA3-KanMX* knockout strains. *TRM10* alleles from the donor strains were amplified by PCR with approximately 200-bp overlapping sequence and introduced into recipient strains to replace *URA3-KanMX* cassette. For *SUP45* replacement *URA3-KanMX* cassette from pCORE was inserted downstream of *SUP45* coding sequence in recipient strains. pCORE cassette was then replaced by the PCR-amplified *SUP45* allele from donor strains. Allele replacements were confirmed by sequencing.

### Sequencing

 The RM *TRM10* and *SUP45* sequences were obtained from the whole genome-sequencing project at the Broad Institute (http://www.broad.mit.edu/annotation/genome/saccharomyces_cerevisiae/Home.html).

All sequencing was done using standard dideoxy methods.

### Dual luciferase assay

Dual luciferase assay was performed as explained before [19]. Dual luciferase reporter plasmids were kindly provided by David Bedwell. Plasmids with the stop codon (pDB691) or the sense codon (pDB690) were transformed into the indicated yeast strains, and transformants were selected on SMD drop-out plates lacking uracil. Transformed strains were grown in liquid SMD medium to a cell density of 0.5–0.7 A600 units/mL as measured using Synergy 2 Multi-Mode Microplate Reader (BioTek Instruments). The luciferase assay was performed using the Dual-Luciferase Reporter Assay System (Promega; E1910). Approximately 10^4^ yeast cells from each strain expressing the indicated dual luciferase reporter were lysed using 100 µL of Passive Lysis Buffer in a 96-well plate (Costar; 3370). Two microliters of the lysate were added to 10 µL of the Luciferase Assay Reagent II in an opaque 96-well plate (Costar; 3614). Relative luminescence units (RLUs) produced by firefly luciferase activity were then measured for 10 seconds using Synergy 2 Multi-Mode Microplate Reader (BioTek Instruments). 10 µL of Stop&Glo buffer was then added to quench the firefly activity and activate the *Renilla* luciferase activity. RLUs were again measured for 10 seconds to determine the *Renilla* luciferase activity. Negative controls that contained all the reaction components except cell lysates were used to determine the background for each luciferase reaction and were subtracted from the experimental values obtained. Percent readthrough is expressed as the mean ± the standard deviation of values obtained from at least eight independent dual luciferase assay including at least four biological replicates.

### X-QTL

All X-QTL experiments were done in duplicates. MAT**a** haploid segregants from the indicated cross were selected as explained before [16]. To create the segregating pool, a single colony of the diploid progenitor was inoculated into 5 mL YPD and grown to stationary phase. The diploid culture was spun down and the supernatant was decanted. The diploid pellet was then resuspended in 50 mL SPO++ sporulation medium. The sporulation was kept at room temperature (∼22°C) with shaking and monitored for the fraction of diploids that had sporulated. Once more than 50% of the diploids had sporulated, 10 mL of the sporulation were spun down and then the supernatant was decanted. The pellet was resuspended in 2 mL water. 600 µL β-glucoronidase (Sigma; G7770) were added to the preparation, and the mixture was incubated at 30°C for one hour. Water was added to the sample so that the total volume was 20 mL. The spore preparation was spread onto SMD + canavanine/thialysine plates (Sigma; C9758 for canavanine (L-canavanine sulphate salt); A2636 for thialysine (S-(2-aminoethyl)- L-cysteine hydrochloride)), with 100 µL of sample going onto each plate. The plates were incubated at 30°C for two days. Then 10 mL of water were poured onto each plate and a sterile spreader was used to remove the segregants from the plate. The cell mixtures from each plate were then pipetted off the plates into a container. The pool was spun down and the water decanted. Haploid segregants were then inoculated into liquid SMRaf plus canavanine medium at a concentration of ∼1×10^7^ cells mL^−1^. The cells were grown for approximately two generations to a density of ∼2×10^7^ cells mL^−1^. To induce the GAL promoter, cells were spun down and then were transferred to liquid SMGal plus canavanine with a density of ∼2×10^6^ cells mL^−1^and were incubated in 30°C while shaking on a rotary shaker at 200 rpm for four hours. Cells were then sorted using BD FACS Vantage SE, collecting 20,000 cells from top 1% (High GFP) and bottom 1% (Low GFP) of the whole population. High GFP, Low GFP and a sample of the whole population were then plated on YPD plates and were incubated at 30°C for two days. DNA was extracted from the grown cells using Genomic-tip 100/G columns (Qiagen; 10243). DNA was labeled using the BioPrime Array CGH Genomic Labeling Module (Invitrogen; 18095-012) with the sample being labeled with Cy3 dUTP and the reference being labeled with Cy5 dUTP. We used a BY/RM diploid as the reference for all hybridizations. Labeled samples were then hybridized onto the allele-specific genotyping microarray with isothermal probes that assays ∼18,000 single nucleotide polymorphisms (SNPs) between BY and RM [16]. Hybridization intensities were extracted and normalized using the rank invariant method in the Agilent Feature Extraction software package. For a given SNP, the difference in the log_10_ ratios of BY and RM-specific probes on a single array (or log_10_ intensity difference) was computed. Background allele frequency changes that occur during pool construction were removed from the data for top 1% (High GFP) and bottom 1% (Low GFP) selection by subtracting the log_10_ intensity difference obtained for the whole (unselected) population from the log_10_ intensity difference observed in the High and Low GFP selections. These steps were conducted in R (http://www.r-project.org/).

## Supporting Information

Figure S1 Region corresponding to allele frequency skew on chromosome XV. 30 kb surrounding the region corresponding to the allele frequency skew on chromosome XV and the genes residing in this region is shown.
(TIF)

Figure S2 Region corresponding to allele frequency skew on chromosome II. 30 kb surrounding the region corresponding to the allele frequency skew on chromosome II and the genes residing in this region is shown.
(TIF)

Figure S3 Comparing the effects of *SUP45* coding polymorphisms and *SUP45* regulatory polymorphisms on readthrough. The effect of swapping *SUP45* coding sequence (white bars) with the alternative allele on readthrough is compared to swapping *SUP45* coding and upstream sequence (grey bars) with the alternative allele in A) BY background and B) RM background.
(TIF)

Figure S4 Distribution of linkage disequilibrium for 1000 random SNP pairs in *S. cerevisiae* population. 1000 random SNP pairs were chosen to have approximately similar frequencies as *TRM10* and *SUP45*. Each pair consists of SNPs from two different chromosomes. The red arrow shows r^2^ for *TRM10* and *SUP45* pair.
(TIF)

Table S1 Comparing percent readthrough measured with the dual luciferase assay and the GFP reporter. Percent readthrough measured using dual luciferase reporter system (as defined in the text) is compared to percent readthrough measured using the GFP reporter (as defined in the text) for BY and RM.
(DOC)

Table S2 Grouping *S. cerevisiae* strains based on their *SUP45* and *TRM10* genotypes. *S. cerevisiae* strains from [18] are grouped based on their *SUP45* and *TRM10* genotypes. For example, strains with the BY allele of *SUP45* are listed under *SUP45* ^BY^.
(DOC)
