# Supplementary figures and images for: Variants in SUP45 and TRM10 Underlie Natural Variation in Translation Termination Efficiency in Saccharomyces cerevisiae
Source: PLoS Genet. 2011 Jul 28;7(7):e1002211. doi: 10.1371/journal.pgen.1002211 (PMC3145625; doi:10.1371/journal.pgen.1002211)

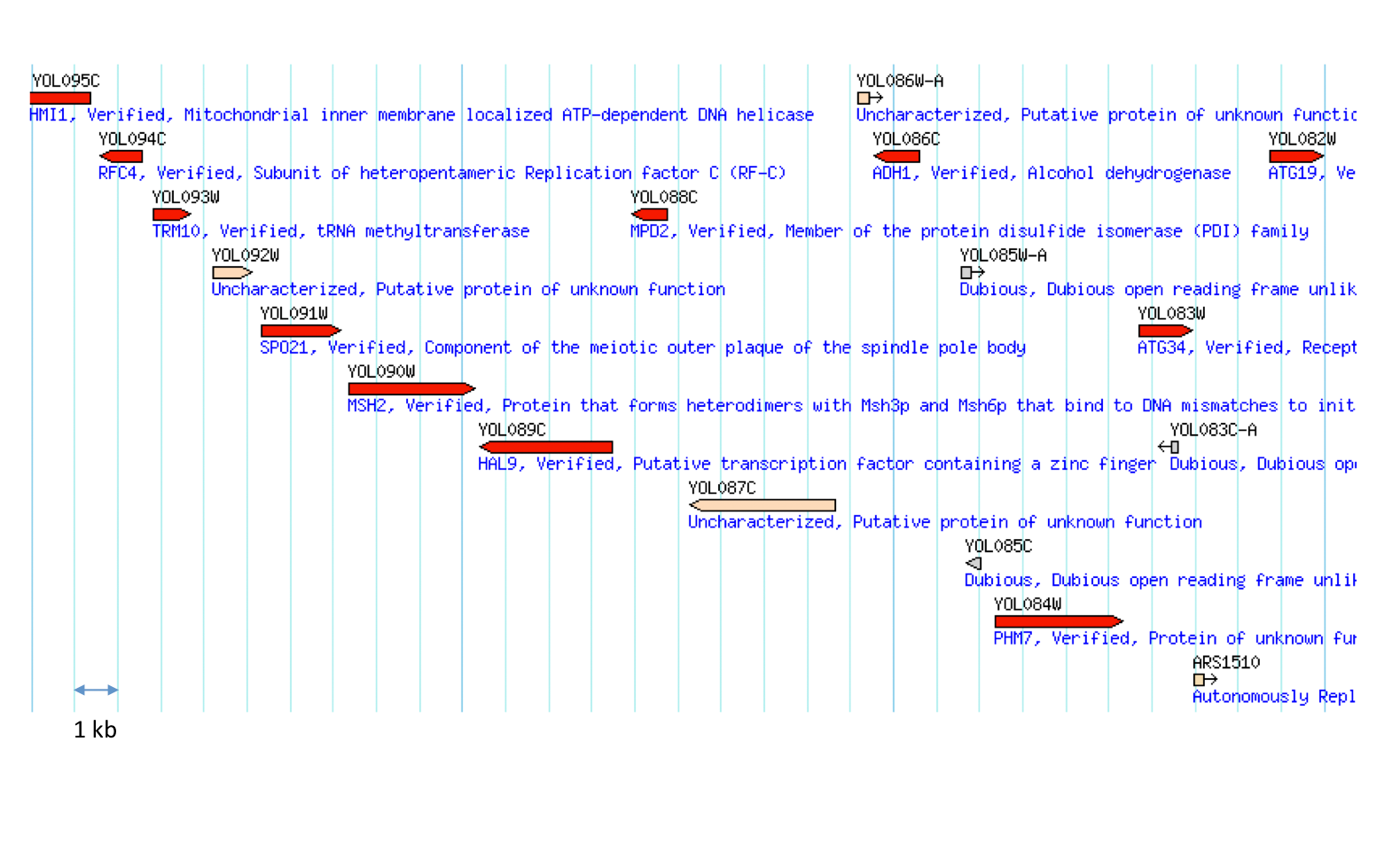

Supplement: Figure S1 — Region corresponding to allele frequency skew on chromosome XV. 30 kb surrounding the region corresponding to the allele frequency skew on chromosome XV and the genes residing in this region is shown. (TIF) [file pgen.1002211.s001.tif]

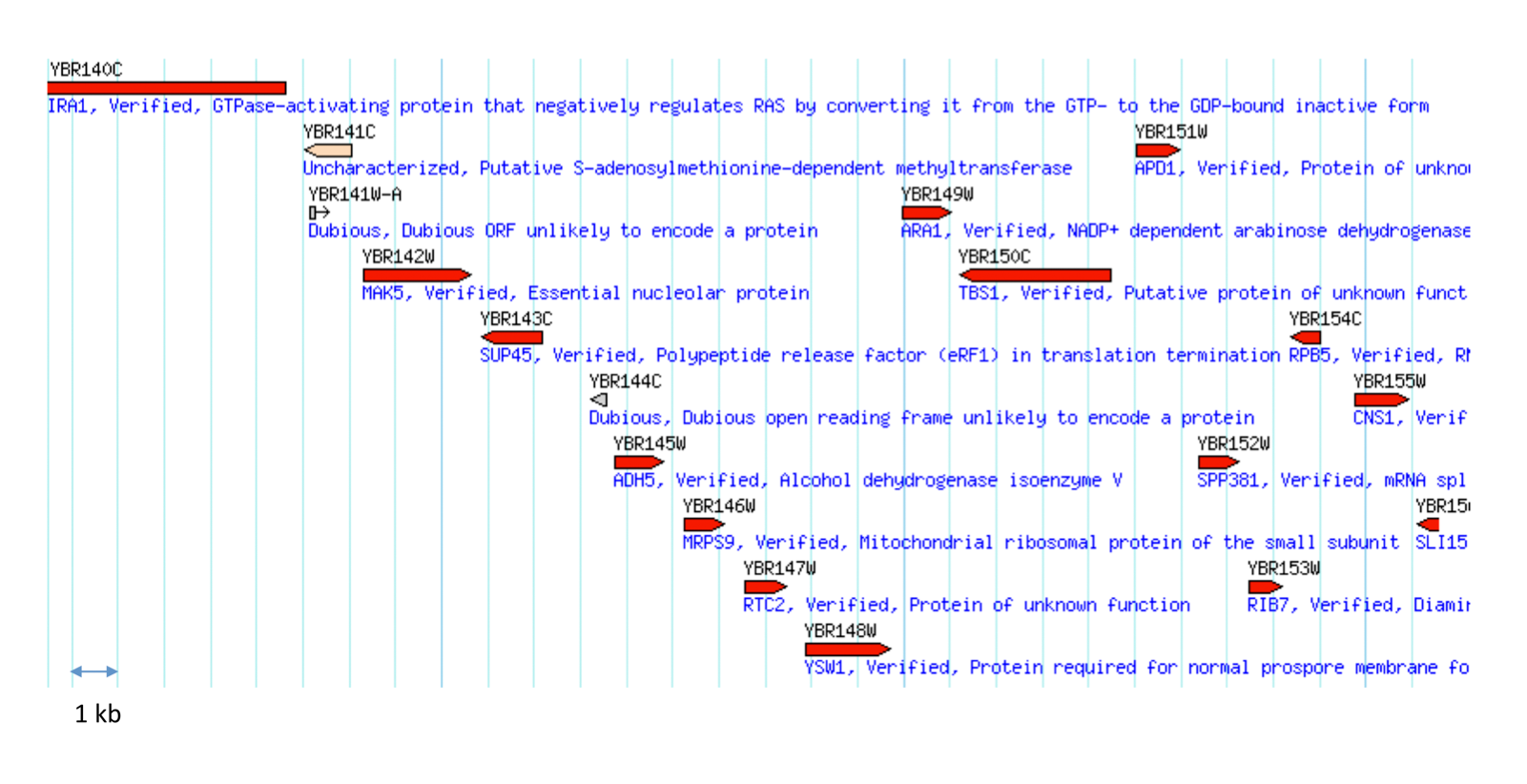

Supplement: Figure S2 — Region corresponding to allele frequency skew on chromosome II. 30 kb surrounding the region corresponding to the allele frequency skew on chromosome II and the genes residing in this region is shown. (TIF) [file pgen.1002211.s002.tif]

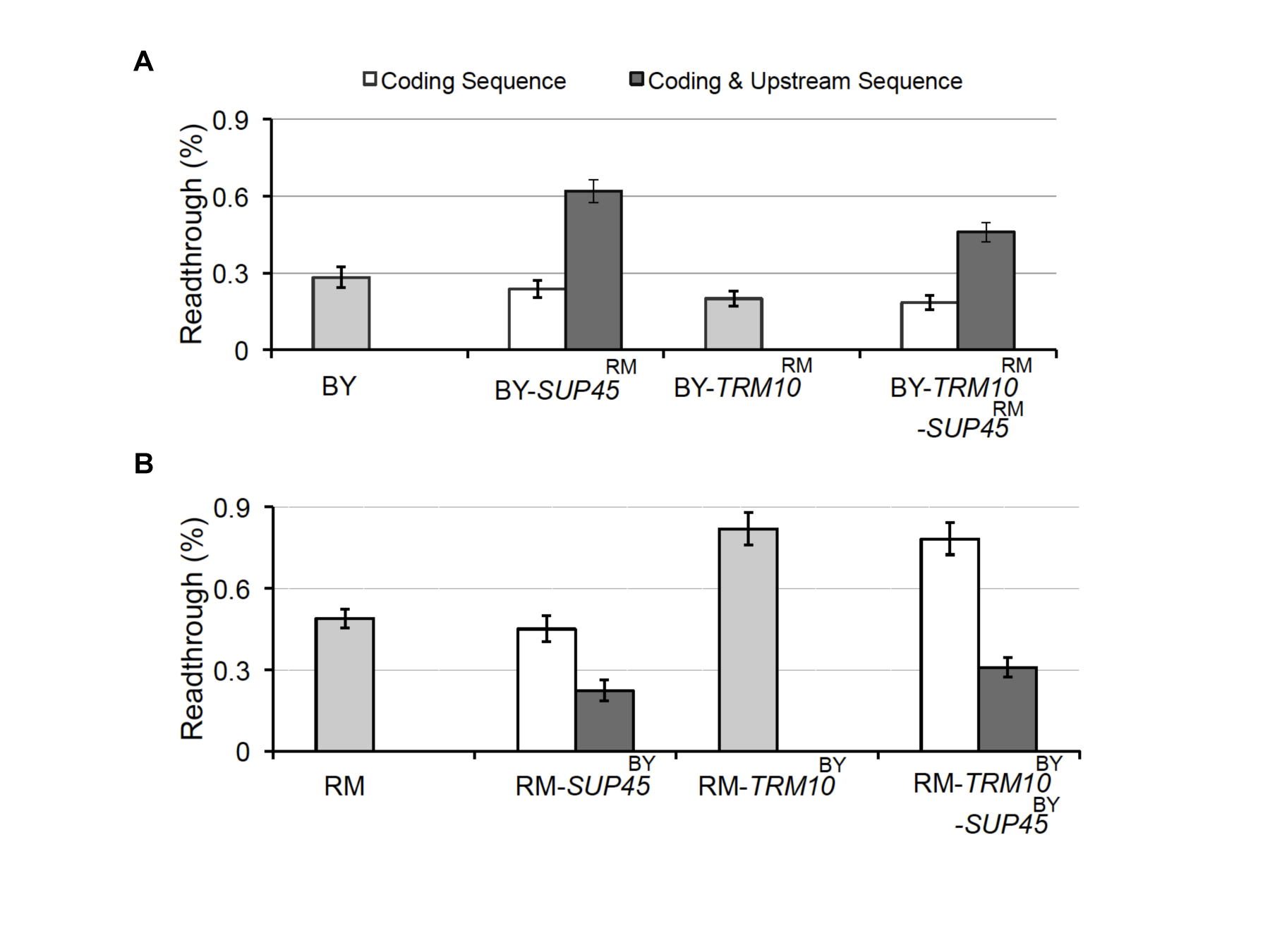

Supplement: Figure S3 — Comparing the effects of SUP45 coding polymorphisms and SUP45 regulatory polymorphisms on readthrough. The effect of swapping SUP45 coding sequence (white bars) with the alternative allele on readthrough is compared to swapping SUP45 coding and upstream sequence (grey bars) with the alternative allele in A) BY background and B) RM background. (TIF) [file pgen.1002211.s003.tif]

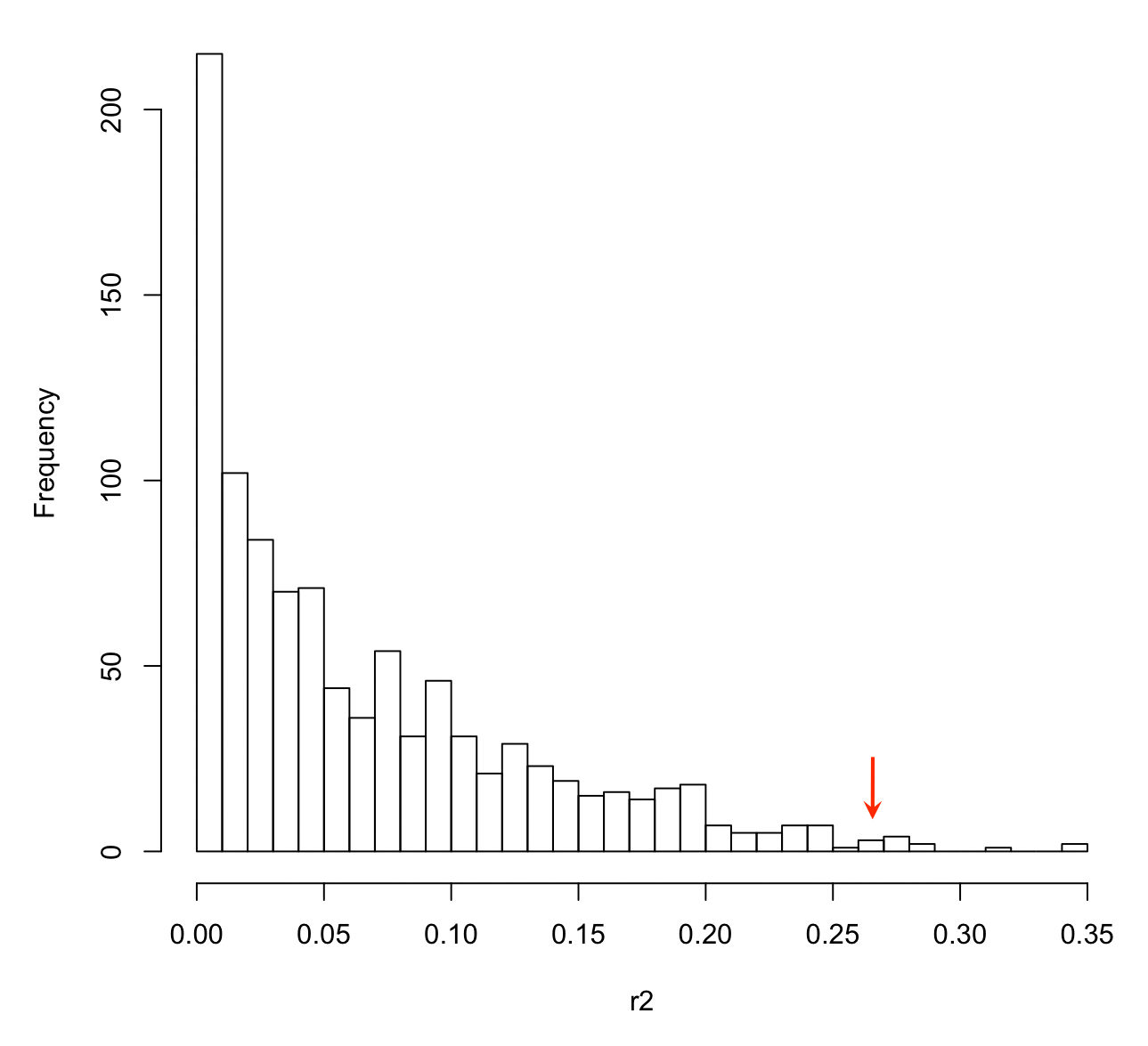

Supplement: Figure S4 — Distribution of linkage disequilibrium for 1000 random SNP pairs in S. cerevisiae population. 1000 random SNP pairs were chosen to have approximately similar frequencies as TRM10 and SUP45. Each pair consists of SNPs from two different chromosomes. The red arrow shows r2 for TRM10 and SUP45 pair. (TIF) [file pgen.1002211.s004.tif]
