# Supplementary material for: Variants in SUP45 and TRM10 Underlie Natural Variation in Translation Termination Efficiency in Saccharomyces cerevisiae
Source: PLoS Genet. 2011 Jul 28;7(7):e1002211. doi: 10.1371/journal.pgen.1002211 (PMC3145625; doi:10.1371/journal.pgen.1002211)
Supplement: Table S1 — Comparing percent readthrough measured with the dual luciferase assay and the GFP reporter. Percent readthrough measured using dual luciferase reporter system (as defined in the text) is compared to percent readthrough measured using the GFP reporter (as defined in the text) for BY and RM. (DOC) [file pgen.1002211.s005.doc]

**Table S1.** **Comparing percent readthrough measured with the dual luciferase assay and the GFP reporter.**

| **Strain** | **Dual luciferase assay** | **GFP reporter** |
| --- | --- | --- |
| BY | 0.272 ± 0.0409 | 2.23 ± 0.720 |
| RM | 0.489 ± 0.0371 | 4.18 ± 0.953 |
