# Supplementary material for: Variants in SUP45 and TRM10 Underlie Natural Variation in Translation Termination Efficiency in Saccharomyces cerevisiae
Source: PLoS Genet. 2011 Jul 28;7(7):e1002211. doi: 10.1371/journal.pgen.1002211 (PMC3145625; doi:10.1371/journal.pgen.1002211)
Supplement: Table S2 — Grouping S. cerevisiae strains based on their SUP45 and TRM10 genotypes. S. cerevisiae strains from [18] are grouped based on their SUP45 and TRM10 genotypes. For example, strains with the BY allele of SUP45 are listed under SUP45 BY. (DOC) [file pgen.1002211.s006.doc]

Table S2. Grouping *S. cerevisiae* strains based on their *SUP45* and *TRM10* genotypes.

| ***SUP45^BY^*** | ***SUP45^RM^*** | ***TRM10^BY^*** | ***TRM10^RM^*** |
| --- | --- | --- | --- |
| 322134S | 273614N | 322134S | 273614N |
| 378604X | CECT10109 | 378604X | CECT10109 |
| A364A | CLIB154 | A364A | CLIB154 |
| BY | CLIB157 | BY | CLIB157 |
| CENPK | CLIB208 | CENPK | CLIB192 |
| CLIB192 | CLIB272 | CLIB219 | CLIB208 |
| CLIB219 | CLIB294 | CLIB318 | CLIB272 |
| CLIB413 | CLIB318 | CLIB324 | CLIB294 |
| EM93 | CLIB324 | CLIB413 | CLIB382 |
| FL100 | CLIB382 | DBVPG1853 | CLIB483 |
| I14 | CLIB483 | EM93 | DBVPG1373 |
| K1 | DBVPG1373 | FL100 | DBVPG1399 |
| K12 | DBVPG1399 | K1 | DBVPG1788 |
| TL229 | DBVPG1788 | K12 | DBVPG1794 |
| W303 | DBVPG1794 | SK1 | DBVPG3591 |
| Y12 | DBVPG1853 | W303 | DBVPG4651 |
| Y3 | DBVPG3591 | Y12 | I14 |
| Y6 | DBVPG4651 | Y55 | M22 |
| Y9 | M22 | Y6 | RM11 |
| YJM145 | RM11 | Y9 | T73 |
| YJM320 | SK1 | YJM145 | TL229 |
| YJM326 | T73 | YJM269 | UC1 |
| YJM413 | UC1 | YJM280 | UC8 |
| YJM436 | UC8 | YJM326 | WE372 |
| YJM454 | WE372 | YJM413 | Y3 |
| YJM653 | Y55 | YJM421 | Y8 |
| YPS1000 | Y8 | YJM454 | Y9J |
| YPS163 | Y9J | YPS1000 | YJM320 |
|  | YJM269 | YPS163 | YJM428 |
|  | YJM280 |  | YJM434 |
|  | YJM421 |  | YJM436 |
|  | YJM428 |  | YJM653 |
|  | YJM434 |  | YJM978 |
|  | YJM978 |  | YJM981 |
|  | YJM981 |  |  |

## 
